# Banxia Xiexin Decoction Ameliorates t-BHP-Induced Apoptosis in Pancreatic Beta Cells by Activating the PI3K/AKT/FOXO1 Signaling Pathway

**DOI:** 10.1155/2020/3695689

**Published:** 2020-04-17

**Authors:** Li-juan Du, Bing Pang, Yu-meng Tan, Ya-nan Yang, Mei-zhen Zhang, Qing Pang, Min Sun, Qing Ni

**Affiliations:** ^1^Department of Endocrinology, Guang'anmen Hospital of China Academy of Chinese Medical Sciences, Beijing, China; ^2^School of Life Sciences, Anhui University, Hefei, China

## Abstract

**Background:**

Banxia Xiexin Decoction (BXXD) reportedly regulates glycolipid metabolism and inhibits pancreatic *β*-cell apoptosis. This study is aimed at investigating the protective effect of BXXD on *tert*-butyl hydroperoxide- (t-BHP-) induced apoptosis in MIN6 cells and the underlying mechanisms.

**Methods:**

MIN6 cells were preincubated with BXXD or liraglutide (Li) with or without PI3K inhibitor LY294002 (LY) for 12 h, following which t-BHP was added to induce MIN6 cell apoptosis. The protective effects of BXXD on MIN6 cells were evaluated by detecting cell viability and proliferation and glucose-stimulated insulin secretion (GSIS). The antiapoptotic effects were evaluated by Hoechst 33342 staining and terminal deoxynucleotidyl transferase dUTP nick end labeling assay (TUNEL). Malondialdehyde and glutathione peroxidase content and superoxide dismutase activity were measured using commercial kits. The expression of PI3K/AKT/FOXO1 signaling pathway-related signal molecules, and that of apoptotic indicators Bax, P27, and Caspase-3, was quantified using western blotting.

**Results:**

Preincubation with BXXD significantly improved t-BHP-induced proliferation inhibition and apoptosis and enhanced GSIS. t-BHP induced the generation of reactive oxygen species and inhibited the activities of antioxidant enzymes, which could be neutralized by pretreatment with BXXD. BXXD promoted the phosphorylation of AKT and FOXO1 in t-BHP-induced MIN6 cells. Moreover, BXXD attenuated the expression of related apoptotic indicators Bax, P27, and Caspase-3. LY abolished these effects of BXXD.

**Conclusion:**

BXXD protected MIN6 cells against t-BHP-induced apoptosis and improved insulin secretory function through modulation of the PI3K/AKT pathway and the downstream FOXO1, thus suggesting a novel therapeutic approach for type 2 diabetes mellitus (T2DM).

## 1. Introduction

The dramatic increase in the incidence of diabetes mellitus (DM) is becoming a major public health issue [1]. China reported 415 million patients with diabetes in 2015, and it is estimated that this number will reach 640 million by the year 2040 [2]. Diabetic complications are severe and irreversible, dramatically affecting the quality of life of the patients. Moreover, the expenses of diabetic vascular complications account for 80% of the total direct medical expenses, resulting in a large economic burden for society [3–5]. Therefore, the development of strategies for early prevention and treatment is necessary.

Pancreatic *β*-cell apoptosis plays a vital role in the development of T2DM [6]. Enhanced pancreatic *β*-cell apoptosis leads to *β*-cell loss in T2DM, which is characterized by progressive deterioration and ultimate failure of *β*-cell function. Reversal of *β*-cell function is critical for islet function, such as in maintenance of healthy glucose metabolism [7, 8]. Oxidative stress damage may be an important factor resulting in pancreatic *β*-cell apoptosis. The PI3K/AKT signaling pathway, as a classical signaling pathway, plays an important role in the growth, proliferation, differentiation, and metabolism of pancreatic *β*-cells. PI3K, stimulated by a series of upstream molecules, acts on the downstream target of AKT and regulates its phosphorylation, leading to alterations in the expression of a protein belonging to the Bcl-2 family and the activation of caspase, which plays an important role in regulating *β*-cell apoptosis [9–12].

Banxia Xiexin Decoction is a well-known traditional Chinese herbal formula containing seven herbs and is commonly used to treat gastrointestinal diseases, diabetes, and related diseases [13–16]. Our previous studies demonstrated that BXXD lowers blood glucose, inhibits pancreatic *β*-cell apoptosis, attenuates oxidative stress, and promotes glucagon-like peptide 1 secretion [17, 18]. However, the mechanisms underlying the action of BXXD have not been elucidated. In this study, we aimed to explore the mechanisms by which BXXD modulates pancreatic *β*-cell apoptosis and insulin secretion through the PI3K/AKT/FOXO1 signaling pathway, thus providing novel scientific evidence for traditional Chinese medicine therapy.

## 2. Materials and Methods

### 2.1. Cell Culture

Mouse MIN6 pancreatic *β*-cells were obtained from the American Type Culture Collection (ATCC). MIN6 cells were grown in high-glucose Dulbecco's modified Eagle's medium (DMEM) (HyClone, USA) containing 25 mmol/l glucose supplemented with 15% fetal bovine serum (FBS) (Lonza, Switzerland), 100 U/ml penicillin, 100 *μ*g/ml streptomycin, and 5 *μ*l/l *β*-mercaptoethanol and cultured in a humidified atmosphere containing 5% CO_2_ at 37°C.

### 2.2. MTT Assay

The cytotoxicity of MIN6 cells was determined by a 3-(4,5-dimethylthiazol-2-yl)-2,5-diphenyltetrazolium bromide (MTT) assay. MIN6 cells were seeded into 96-well plates at a density of 5 × 10^3^ cells/well. For determining the cytotoxicity of BXXD, the cells were pretreated with different concentrations of BXXD (0.1, 0.5, 1, 1.5, 2.5, 5, and 10 mg/ml) for 12 h. Subsequently, 100 *μ*M t-BHP was added, followed by incubation for 2 h. Then, the MTT solution (0.5 mg/ml) was added to each well, followed by incubation for 4 h at 37°C. The supernatant was discarded, 150 *μ*l Dimethyl sulfoxide (DMSO) for each well, and the cells were shaken for 15 min. Absorbance (OD value) at a wavelength of 490 nm was measured using a microplate reader. MIN6 cell viability (%) was calculated based on the absorbance of the treatment sample group/absorbance of the control group × 100.

### 2.3. Cell Apoptosis Assay

MIN6 cell apoptosis was detected using the chromatin-specific dye Hoechst 33342 (Sigma, Germany) staining and TUNEL (Shanghai Beyotime, China) assay. After pretreatment with BXXD (0.5 mg/ml), Li (50 Nm), LY (100 *μ*M), Li+LY, or BXXD+LY for 12 h, 100 *μ*M t-BHP (Tianjin Alfa Aesar, China) was added, followed by incubation for 2 h. The cells were then washed three times with phosphate-buffered saline (PBS) (HyClone, USA). For Hoechst staining, 5 *μ*g/ml Hoechst was added, followed by incubation for 20 min. The dye solution was then allowed to absorb, followed by washing three times with PBS. For the TUNEL staining, MIN6 cells were fixed with a 4% paraformaldehyde solution for 30 min at room temperature and then permeabilized with 0.3% Triton X-100 for 5 min. Following a wash with PBS, samples were first incubated with terminal deoxynucleotide TUNEL reagent containing terminal deoxynucleotidyl transferase and fluorescein isothiocyanate-dUTP. The cells were then counterstained with 1 *μ*g/ml 4′,6-diamidino-2-phenylindole (DAPI) for 30 min. Images were taken using a fluorescence microscope.

### 2.4. Glucose-Stimulated Insulin Secretion (GSIS)

MIN6 cells were seeded in 24-well plates in triplicate, treated with BXXD, Li, or LY for 12 h. 100 *μ*M t-BHP was added, followed by incubation for 2 h. Cells were preincubated in 0.5 ml Krebs-Ringer bicarbonate (KRB) buffer containing 2.8 mM glucose for 1 h. The KRB buffer was removed and replaced by 0.5 ml KRB buffer containing 2.8 mM glucose for 1 h (this was considered the basal level). The islets were then incubated in KRB buffer containing 16.7 mM glucose for 1 h (this was considered the stimulated level). Insulin contents were determined using a mouse insulin ELISA kit (Mercodia, Uppsala, Sweden). The insulin stimulatory index of islets was calculated as stimulated/basal insulin secretion.

### 2.5. Estimation of Oxidative Stress Parameters (MDA, SOD, and GSH-Px)

For the measurement of malondialdehyde (MDA), cells were grown in flasks. After treatment with the indicated drugs for 12 h, the cells were subsequently treated with 100 *μ*M t-BHP for 2 h. Then, the cells were harvested and lysed for the detection of MDA content. For detecting the activity of superoxide dismutase (SOD) and glutathione peroxidase (GSH-Px), cells were seeded in 24-well plates and treated as mentioned above. The culture media were collected for SOD and GSH-Px assays. The MDA, SOD, and GSH-Px levels were measured using commercial kits (Nanjing Jiancheng, China) according to the manufacturer's instructions. The protein concentration of the cell lysates was determined using a bicinchoninic acid (BCA) assay kit. The MDA content was normalized by the cellular protein content.

### 2.6. Intracellular Reactive Oxygen Species (ROS) Detection

Intracellular ROS levels were measured using a dichloro-dihydro-fluorescein diacetate (DCFH-DA) probe (Sigma, Germany). MIN6 cells (5 × 10^3^) were seeded in quadruplicate into a 96-well plate and treated as mentioned above. Cells were incubated in DMEM (phenol red-free) containing 10 *μ*M DCFH-DA at 37°C for 30 min and then washed three times with PBS to remove the residual probe. The cells were then washed with DMEM without phenol red. The fluorescence intensities were detected using a fluorescence microplate reader (BioTek, USA) at Ex 485/Em 530 nm.

### 2.7. Western Blotting

After treatment, MIN6 cells were harvested, washed with cold PBS 3 times, and incubated in RIPA lysis buffer on ice for 20 min. The lysis conditions were as follows: 12000 rpm at 4°C. Protein content was quantified using a BCA assay kit (Thermo Scientific, China). After the loading buffer was added and mixed, the sample was boiled for 10 min, cooled, and stored in a -20°C refrigerator. Aliquots were separated by 10% sodium dodecyl sulfate-polyacrylamide gel electrophoresis (SDS-PAGE) and then transferred to polyvinylidene fluoride membranes (Bio-Rad, USA). Membranes were blocked using SuperBlock T20 (TBS) buffer for 1 h, then incubated with primary antibodies overnight at 4°C, and washed three times with TBST. The dilution ratio used was 1 : 500, and the proteins were as follows: p27 (Cell Signaling Technology, USA), AKT (Cell Signaling Technology, USA), AKT (Phospho-Ser473) (Cell Signaling Technology, USA), FOXO1 (Cell Signaling Technology, USA), FOXO1 (phospho Ser256) (Cell Signaling Technology, USA), Bax (Proteintech, USA), and Caspase-3 (Proteintech, USA). Quantification of protein expression was normalized to that of *β*-actin (Shanghai Abmart, China) (1 : 5000). The membranes were then incubated with horseradish peroxidase-conjugated secondary antibodies for 2 h at room temperature. The FluorChem™ E System (ProteinSimple, USA) was used to visualize bands of the antigen-antibody complexes, and the ImageJ software (NIH, USA) was employed for densitometric analysis to obtain band intensities.

### 2.8. Statistical Analysis

All data were representative of at least three separate experiments. The values were expressed as mean ± SEM, and images were presented by GraphPad Prism 7.0 Software (GraphPad Software, USA). A one-way analysis of variance (ANOVA) with the Bonferroni test and Tukey test was used to compare between groups. SPSS version 20.0 (IBM Corp., Armonk, NY, USA) was used for the analyses. Values of *P* < 0.05 was considered to be statistically significant.

## 3. Results

### 3.1. Cytotoxicity and Cell Proliferation in MIN6 Cell Exposed to BXXD

**Figure 1:**
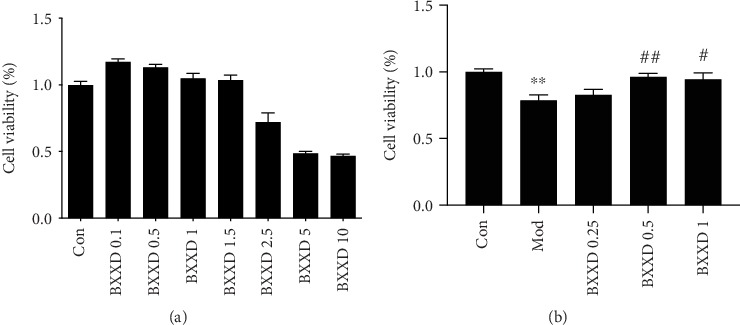
Determination of BXXD experiment conditions. (a) MIN6 cells were preconditioned with different concentrations of BXXD (0.1, 0.5, 1, 1.5, 2.5, 5, and 10 mg/ml) for 12 h. (b) MIN6 cells were pretreated with BXXD (0.25, 0.5, and 1 mg/ml) for 12 h and then treated with 100 *μ*M t-BHP for 2 h. Cell viability was detected by MTT assay. MIN6 cells were incubated with DMEM (Con), DMEM plus t-BHP (Mod), and DMEM, t-BHP plus different concentrations of BXXD (BXXD 0.25, BXXD 0.5, and BXXD 1). Values are expressed as mean ± SEM. The experiment was repeated three times. ^∗∗^*P* < 0.01 versus Con; ^∗^*P* < 0.05 versus Con; ^##^*P* < 0.01 versus Mod, and ^#^*P* < 0.05 versus Mod.

No evident cytotoxic effects of BXXD were observed at doses of 0.1-1.5 mg/ml; BXXD reduced cell viability from 2.5 to 10 mg/ml (Figure 1(a)). Therefore, we further studied whether BXXD could reverse t-BHP-induced cell proliferation inhibition. Cells were preincubated with different concentrations of BXXD (0.25, 0.5, and 1 mg/ml) and then stimulated with 100 *μ*M t-BHP for 2 h. As shown (Figure 1(b)), t-BHP stimulation significantly reduced cell proliferation, and BXXD preincubation increased cell proliferation in an approximately dose-dependent manner. Therefore, the concentration of 0.5 mg/ml was used in subsequent experiments.

### 3.2. BXXD Protected MIN6 Cells by Reducing MIN6 Cell Apoptosis

**Figure 2:**
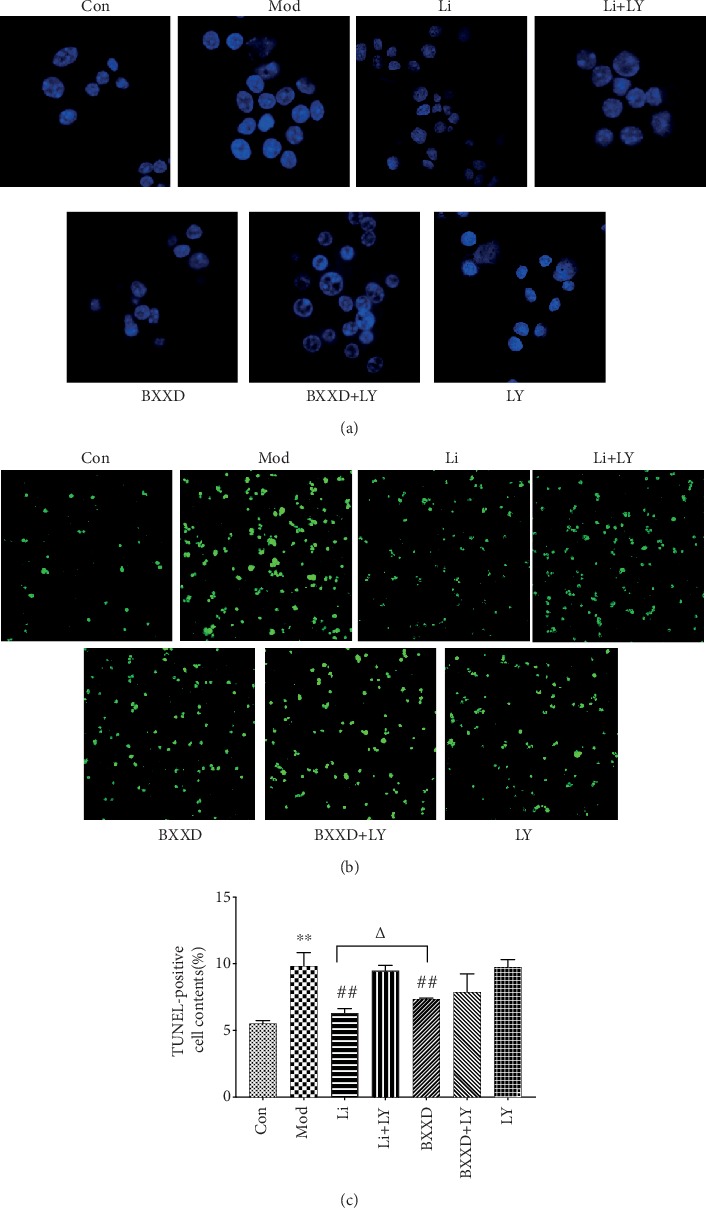
BXXD pretreatment attenuates t-BHP-induced apoptosis in MIN6 cells. Morphology of t-BHP-induced apoptosis in MIN6 cells pretreated with BXXD observed by (a) TUNEL and Hoechst staining and by (b) Hoechst staining observed under a fluorescence microscope. (c) The percentage of apoptotic cells in each group by TUNEL. MIN6 cells were incubated with DMEM (Con), DMEM plus t-BHP (Mod), DMEM, t-BHP plus liraglutide (Li), DMEM, t-BHP, liraglutide plus LY294002 (Li+LY), DMEM, t-BHP plus BXXD 0.5 mg/ml (BXXD), and DMEM, t-BHP, BXXD 0.5 mg/ml plus LY294002 (BXXD+LY). Values are expressed as mean ± SEM. The experiment was repeated three times. ^∗^*P* < 0.05 versus control group; ^∗∗^*P* < 0.01 versus control group; ^#^*P* < 0.05 versus model group; ^##^*P* < 0.01 versus model group; ^△^*P* < 0.05 BXXD versus Li group; and ^△△^*P* < 0.01 BXXD versus Li group.

Hoechst 33342 staining revealed that cells of the control group were intact and their nuclei were evenly stained. After t-BHP stimulation, the cells were irregularly condensed and agglomerated, showing dense staining and the nucleus splitting into fragments. Compared with the model group, the BXXD and liraglutide group showed alleviated nuclear condensation and reduced cell debris. Besides, when LY294002 was added, the protective effect of BXXD and liraglutide was not found to be ideal (Figures 2(a) and 2(b)). t-BHP induction markedly induced apoptosis in MIN6 cells; the percentage of apoptotic cells markedly increased in the model group compared to the control group (9.84 ± 1.00% versus 5.50 ± 0.22%, *P* < 0.05). Cotreatment of MIN6 cells with 100 *μ*M t-BHP and BXXD (0.5 mg/ml) for 12 h reduced the percentage of apoptotic cells to 7.35 ± 0.08% (*P* < 0.05). The positive cell content rate for the liraglutide group was 6.31 ± 0.32% (*P* < 0.05). A significant difference was found between the BXXD group and the liraglutide group (*P* < 0.05). In addition, when LY294002 was preadded, BXXD and liraglutide could not improve the apoptosis induced by t-BHP (Figure 2(c)).

### 3.3. BXXD Rectified High-Glucose-Stimulated Insulin Secretion

**Figure 3:**
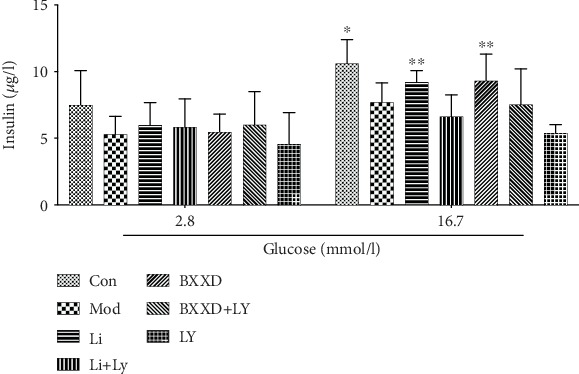
Effect of BXXD on insulin secretion. MIN6 cells were incubated with DMEM (Con), DMEM plus t-BHP (Mod), DMEM, t-BHP plus liraglutide (Li), DMEM, t-BHP, liraglutide plus LY294002 (Li+LY), DMEM, t-BHP plus BXXD 0.5 mg/ml (BXXD), and DMEM, t-BHP, BXXD 0.5 mg/ml plus LY294002 (BXXD+LY). ^∗^*P* < 0.05 versus 2.8 mM Glc; ^∗∗^*P* < 0.01 versus 2.8 mM Glc.

To investigate the effect of BXXD on *β*-cell function, insulin secretion in response to glucose stimulation was detected. GSIS was impaired in t-BHP-treated MIN6 cells. As shown in Figure 3, 16.7 mM glucose failed to promote insulin release in the model group compared to the 2.8 mM group (7.70 ± 1.46 at 16.7 mM Glc versus 5.29 ± 1.35 at 2.8 mM Glc, *P* > 0.05). However, BXXD and liraglutide could effectively release insulin compared to that observed in the model group (BXXD: 9.31 ± 1.99 at 16.7 mM Glc versus 5.47 ± 1.33 at 2.8 mM Glc; Li: 9.22 ± 0.86 at 16.7 mM Glc versus 5.98 ± 1.46 at 2.8 mM Glc, *P* < 0.05, respectively), indicating that the GSIS impairment by t-BHP can be reversed by BXXD and liraglutide. In addition, when LY294002 was preadded, the protective effects of BXXD and Li could be neutralized and the GSIS impairment could not be rectified.

### 3.4. BXXD Protected MIN6 Cells from t-BHP-Induced Oxidative Stress

**Figure 4:**
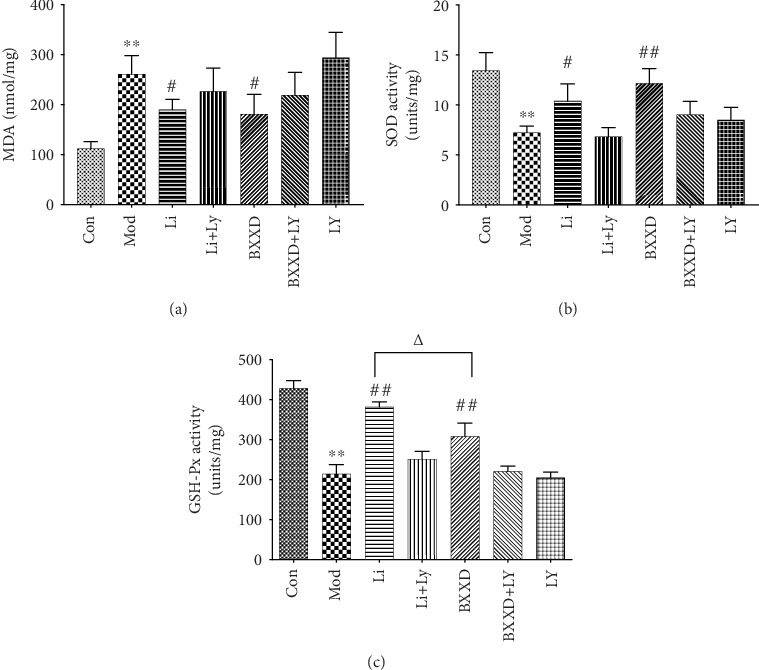
Protective effects of BXXD by regulating antioxidant function by increasing the activities of SOD and GSH-Px and decreasing the production of MDA. MIN6 cells were incubated with DMEM (Con), DMEM plus t-BHP (Mod), DMEM, t-BHP plus liraglutide (Li), DMEM, t-BHP, liraglutide plus LY294002 (Li+LY), DMEM, t-BHP plus BXXD 0.5 mg/ml (BXXD), and DMEM, t-BHP, BXXD 0.5 mg/ml plus LY294002 (BXXD+LY). Values are expressed as mean ± SEM. The experiment was repeated three times. ^∗^*P* < 0.05 versus control group; ^∗∗^*P* < 0.01 versus control group; ^#^*P* < 0.05 versus model group; ^##^*P* < 0.01 versus model group; ^△^*P* < 0.05 BXXD versus Li group; and ^△△^*P* < 0.01 BXXD versus Li group.

To determine whether BXXD affects oxidative stress-related biochemical enzymes, the levels of oxidant and antioxidant enzymes, such as MDA, SOD, and GSH-Px, were measured. t-BHP induction markedly decreased the levels of SOD and GSH-Px and increased the level of MDA (SOD: 7.23 ± 0.64 versus 13.42 ± 1.80, GSH-Px: 214.22 ± 47.26 versus 428.18 ± 39.42, and MDA: 261 ± 37.27 versus 112.12 ± 14.08, *P* < 0.05, respectively). The activities of SOD and GSH-Px were significantly increased in the BXXD pretreatment group relative to the model group (SOD: 12.15 ± 1.48 versus 7.23 ± 0.64, GSH-Px: 308.26 ± 66.19 versus 214.22 ± 47.26, *P* < 0.05, respectively). In contrast, MDA production was significantly decreased compared to that in the model group (181.19 ± 39.29 versus 261 ± 37.27, *P* < 0.05). These results also confirmed that the antioxidant capacity of BXXD was comparable to that of liraglutide (SOD: 12.15 ± 1.48 versus 10.41 ± 1.67, MDA: 181.19 ± 39.29 versus 190.32 ± 20.34, *P* > 0.05). The effect of liraglutide on improving GSH-Px expression was more potent than that of BXXD (308.26 ± 66.19 versus 381.72 ± 25.28, *P* < 0.05). Furthermore, BXXD and liraglutide in the presence of PI3K inhibitor LY294002 could not inhibit oxidative stress more effectively than that in the model group (Figure 4).

### 3.5. BXXD Attenuated Intracellular ROS Generation

**Figure 5:**
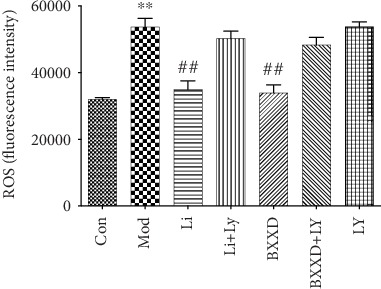
Protective effects of BXXD due to reduced t-BHP-induced generation of intracellular ROS measured by the DCFH-DA assay. MIN6 cells were incubated with DMEM (Con), DMEM plus t-BHP (Mod), DMEM, t-BHP plus liraglutide (Li), DMEM, t-BHP, liraglutide plus LY294002 (Li+LY), DMEM, t-BHP plus BXXD 0.5 mg/ml (BXXD), and DMEM, t-BHP, BXXD 0.5 mg/ml plus LY294002 (BXXD+LY). Values are expressed as mean ± SEM. The experiment was repeated three times. ^∗^*P* < 0.05 versus control group; ^∗∗^*P* < 0.01 versus control group; ^#^*P* < 0.05 versus model group; and ^##^*P* < 0.01 versus model group.

The generation of intracellular ROS induced by t-BHP promotes cellular damage. To determine whether BXXD attenuated cell apoptosis by reducing ROS generation, intracellular ROS concentrations were measured using the DCFH-DA assay. Intracellular ROS generation was significantly increased in t-BHP-treated MIN6 cells compared to nontreated MIN6 cells (53760.75 ± 5057.22 versus 32003.25 ± 1029, *P* < 0.05). However, ROS generation was significantly reduced by BXXD and liraglutide pretreatment (BXXD: 33992.25 ± 4655.02 versus 53760.75 ± 5057.22, Li: 35003 ± 5131.53 versus 53760.75 ± 5057.22, *P* < 0.05, respectively). BXXD and liraglutide in the presence of PI3K inhibitor LY294002 could not decrease ROS generation more effectively than that in the model group (Figure 5).

### 3.6. BXXD Protected MIN6 Cells via Activation of the PI3K/AKT/FOXO1 Pathway

**Figure 6:**
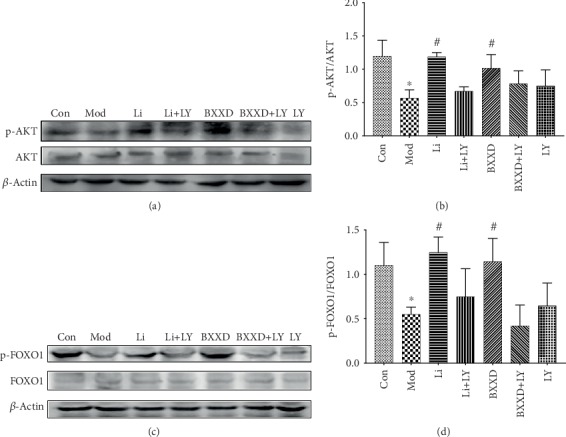
Effects of BXXD on the expression of AKT, phosphorylated AKT, FOXO1, and phosphorylated FOXO1 in MIN6 cells treated with THBP. (a, b) Western blot and quantitative measurement of AKT and phosphorylated AKT in MIN6 cells. Lane loading was normalized by reblotting with *β*-actin. The levels were expressed as p-AKT/AKT and normalized relative to the control group. (c, d) Western blot and quantitative measurement of FOXO1 and phosphorylated FOXO1 in MIN6 cells. Lane loading was normalized by reblotting with *β*-actin. The levels were expressed as p-AKT/AKT and normalized relative to the control group. MIN6 cells were incubated with DMEM (Con), DMEM plus t-BHP (Mod), DMEM, t-BHP plus liraglutide (Li), DMEM, t-BHP, liraglutide plus LY294002 (Li+LY), DMEM, t-BHP plus BXXD 0.5 mg/ml (BXXD), and DMEM, t-BHP, BXXD 0.5 mg/ml plus LY294002 (BXXD+LY). ^∗^*P* < 0.05 versus control group; ^#^*P* < 0.05 versus model group.

To identify the signaling pathway involved in the protective effects of BXXD, we tested the levels of AKT and phospho-AKT proteins in MIN6 cells using western blot. A 2 h incubation with 100 *μ*M t-BHP significantly decreased the phospho-AKT/AKT levels compared with that in the control group (0.57 ± 0.12 versus 1.20 ± 0.24, *P* < 0.05). This decrease was dramatically reversed by BXXD and liraglutide (BXXD: 1.02 ± 0.20 versus 0.57 ± 0.12, Li: 1.19 ± 0.06 versus 0.57 ± 0.12, *P* < 0.05), but no significant difference was found between the BXXD and liraglutide groups (*P* > 0.05). Since FOXO1 is one of the crucial downstream effectors of AKT, we next tested the levels of FOXO1 and phospho-FOXO1 proteins. Phosphorylation of FOXO1 was markedly decreased in the t-BHP group compared to the control group (0.55 ± 0.08 versus 1.10 ± 0.26, *P* < 0.05). Besides, phospho-FOXO1/FOXO1 level was substantially attenuated in the BXXD and liraglutide groups compared to the model group (BXXD: 1.14 ± 0.26 versus 0.55 ± 0.08, *P* < 0.05, liraglutide: 1.25 ± 0.17 versus 0.55 ± 0.08, *P* < 0.05, respectively); however, no discernible difference in phospho-FOXO1/FOXO1 expression was observed between the groups treated with BXXD and liraglutide (*P* > 0.05). When LY294002 was preadded, BXXD and liraglutide could not significantly increase the expression of p-AKT/AKT and p-FOXO1/FOXO1. In addition, there was no significant difference between the control and LY294002-only groups, which ruled out the possible effect of LY294002 (Figure 6).

### 3.7. BXXD Downregulated the Expression of Bax, P27, and Caspase-3

**Figure 7:**
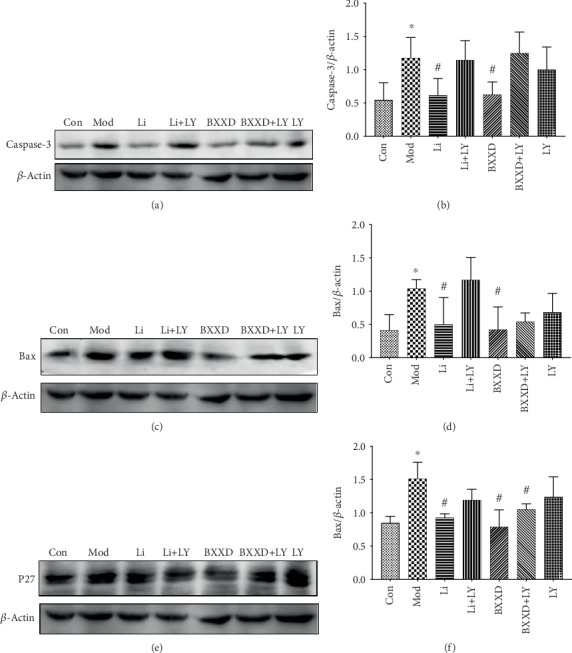
Effects of BXXD on the expression of Bax, P27, and Caspase-3 in MIN6 cells treated with THBP. (a, b) Western blot and quantitative measurement of Caspase-3. (c, d) Western blot and quantitative measurement of Bax. (e, f) Western blot and quantitative measurement of P27. Lane loading was normalized by reblotting with *β*-actin. MIN6 cells were incubated with DMEM (Con), DMEM plus t-BHP (Mod), DMEM, t-BHP plus liraglutide (Li), DMEM, t-BHP, liraglutide plus LY294002 (Li+LY), DMEM, t-BHP plus BXXD 0.5 mg/ml (BXXD), and DMEM, t-BHP, BXXD 0.5 mg/ml plus LY294002 (BXXD+LY). Data was expressed as mean ± SD and shown as a percentage of the control group. ^∗^*P* < 0.05 versus control group; ^#^*P* < 0.05 versus model group.

The proapoptotic Bcl-2 family member Bax and the cell cycle inhibitor P27 have been reported to be immediate target genes of FOXO1. The best recognized biochemical hallmark of both early and late stages of apoptosis is the activation of caspases, and Caspase-3 activation is a critical determinant of apoptosis. Bax, P27, and Caspase-3 expression levels were examined in order to clarify further the molecular mechanism underlying the BXXD-mediated inhibition of apoptosis. The expression of Bax, P27, and Caspase-3 was markedly upregulated in the t-BHP group compared with the control group (Bax: 1.04 ± 0.13 versus 0.41 ± 0.24, P27: 1.51 ± 0.25 versus 0.85 ± 0.10, and Caspase-3: 1.18 ± 0.31 versus 0.55 ± 0.26, *P* < 0.05, respectively). Bax, P27, and Caspase-3 expression levels were markedly downregulated in the BXXD group compared with the t-BHP group (Bax: 0.42 ± 0.34 versus 1.04 ± 0.13, P27: 0.79 ± 0.26 versus 1.51 ± 0.25, and Caspase-3: 0.63 ± 0.19 versus 1.18 ± 0.31, *P* < 0.05, respectively). A noticeable decrease in the expression of Bax, P27, and Caspase-3 was also observed in the liraglutide group compared to the t-BHP group (Bax: 0.50 ± 0.40 versus 1.04 ± 0.13, P27: 0.93 ± 0.06 versus 1.51 ± 0.25, and Caspase-3: 0.62 ± 0.25 versus 1.18 ± 0.31, *P* < 0.05, respectively). No significant difference was found between the BXXD and liraglutide groups (*P* > 0.05). When the PI3K inhibitor LY294002 was preadded, liraglutide could not decrease the expression of Bax, Caspase-3, and P27, and BXXD could not decrease the expression of Bax and Caspase-3 (Figure 7).

## 4. Discussion

BXXD reportedly promotes insulin sensitivity, improves insulin resistance of peripheral target tissues (liver, adipose tissue, and skeletal muscle), and regulates glycolipid metabolism [19–24]. However, the molecular mechanism underlying BXXD effect on *β*-cells is not fully understood. Here, we found that BXXD protected MIN6 pancreatic *β*-cells by inhibiting t-BHP-induced apoptosis and promoting glucose-induced insulin secretion. The mechanisms mediating these effects may be related to the restoration of the PI3K/AKT signaling pathway and its downstream target forkhead box O1 (FOXO1). Additionally, BXXD significantly attenuated oxidative stress and inhibited the expression of related apoptotic indicators Bcl-2-associated X protein (Bax), protein 27 (P27), and cysteine aspartic acid-specific protease 3 (Caspase-3) (Figure 7).

Generally, T2DM is characterized by hyperglycemia and elevated free fatty acid levels, which contribute to the dysfunction and death of pancreatic *β*-cells and lead to insufficient insulin secretion [25, 26]. Oxidative stress damage and multiple-induced caspase cascade may be two crucial factors resulting in pancreatic *β*-cell apoptosis. Persistent hyperglycemia induces ROS formation, leading to increased lipid peroxidation level and/or decreased antioxidase activity, thereby triggering oxidative stress [27, 28]. High levels of ROS and reactive nitrogen produced during oxidative stress can lead to cell dysfunction and death through lipid peroxidation, DNA damage, and cell apoptosis regulation [29]. t-BHP is a short-chain analog of lipid hydroperoxides, which mimics the toxic effect of peroxidized fatty acids. t-BHP has been reported to induce apoptosis in vivo and in vitro [29]. In agreement with previous studies, our results showed that 100 *μ*M t-BHP notably induced apoptosis [30]. Here, we provide evidence that BXXD treatment could inhibit t-BHP-induced apoptosis.

Oxidative stress is marked by the imbalance between the oxidative system and the antioxidant system, toward the oxidative system, which causes damage to the organism [31]. *β*-cell apoptosis lies at the core of DM pathophysiology, and oxidative stress is a mediator of *β*-cell apoptosis [32]. ROS and MDA usually represent oxidative stress indicators, while SOD and GSH-Px are common antioxidant enzymes. Excessive ROS production is closely related to the apoptosis of pancreatic *β*-cells and is the fundamental cause of insulin secretion and utilization disorders [33]. Antioxidant enzymes, such as SOD and GSH-Px, participate in ROS homeostasis. SOD is the first antioxidant enzyme against ROS; it converts O2^−^ into H_2_O_2_, which is further degraded into O_2_ and H_2_O by GSH-Px. GSH-Px catalyzes the hydrogen peroxide reduction by two molecules of glutathione, a part of the ROS defense system. MDA is generated in vivo via the peroxidation of polyunsaturated fatty acids. MDA interacts with proteins and is itself potentially atherogenic. Our study showed an increase in ROS formation and lipid peroxidation in terms of MDA levels and a decrease in antioxidase activity in terms of SOD and GSH-Px in t-BHP-induced MIN6 cells. BXXD significantly attenuated intracellular ROS generation, decreased MDA levels, and increased SOD and GSH-Px levels, which might be one of the mechanisms underlying the attenuation of oxidative stress in MIN6 cells.

The PI3K/AKT signaling pathway plays a pivotal role in regulating cell growth, proliferation, survival, and metabolism. Phosphatidylinositol 3-kinase (PI3K) can be activated by numerous *β*-cell surface receptors, and Protein Kinase B (AKT) is one of the pivotal PI3K effectors [34]. FOXO1 is a key factor located downstream of the PI3K/AKT pathway, and its transcriptional activity is regulated by phosphorylation of AKT [35]. AKT activation is accompanied by phosphorylation of proapoptotic Bad protein and nuclear FOXOs, hence suppression of FOXO1/3a transcription factors, which are known to be important for pancreatic *β*-cell survival [36]. This study investigated the effects of BXXD on t-BHP-induced PI3K/AKT insulin signaling in MIN6 cells and whether BXXD can improve the insulin secretion function of islet *β*-cells through this pathway. The results showed that t-BHP inhibited the serine phosphorylation of AKT and FOXO1 in MIN6 cells. Cotreatment with BXXD reversed the inhibitory effect of t-BHP on AKT and FOXO1 phosphorylation. Furthermore, coadministration of PI3K inhibitor LY294002 with BXXD abolished the BXXD-induced increase in AKT and FOXO1 phosphorylation levels. We also found that BXXD can improve the insulin secretion of islet *β*-cells stimulated by glucose; this effect could also be reversed after the addition of LY294002. This finding suggests that BXXD improves the secretory function of islet *β*-cells by promoting the transduction of the PI3K/AKT/FOXO1 insulin signaling pathway.

FOXO transcription factors can regulate numerous target genes such as cell cycle inhibitor p27, the Bcl-2 protein family [37, 38]. In order to further explore the PI3K/AKT/FOXO1 signaling pathway-related downstream factors and the protective effect of BXXD on MIN6, we screened several apoptosis-related proteins. The Bcl-2 protein family plays a key role in controlling the mitochondrial pathway of *β*-cell apoptosis and the activation of caspases [29]. Bcl-2-related proteins fall into two groups that generally either promote apoptosis (Bax, Bak, and Bad) or repress apoptosis (Bcl-2 and Bcl-xL). Bax is a critical regulator of the mitochondrial apoptotic pathway. Increased Bax expression, which upregulates Caspase-3, was recently reported to correlate with *β*-cell apoptosis. P27, a member of the cyclin-dependent kinase inhibitor (CDKI) family, is highly expressed in *β*-cell nuclei and plays essential roles during G1-to-S phase progression. Moreover, p27-deleted mice displayed improved glucose tolerance and increased insulin secretion, which was attributed to the increased islet mass [39]. In the current study, when apoptosis occurred, the gene expression of Bax, P27, and Caspase-3 increased significantly. Furthermore, when BXXD attenuated the apoptosis, the expression of Bax, P27, and Caspase-3 decreased, indicating that these related apoptotic indicators may be involved in the process by which BXXD protects cells against ROS-induced oxidative damage.

This study has some limitations. The molecular targets of BXXD may not be only dependent on the PI3K/AKT/FOXO1 signaling pathway; the possibility of other mechanisms cannot be excluded. Further studies are required to explore the mechanism of action of BXXD in detail.

## 5. Conclusions

**Figure 8:**
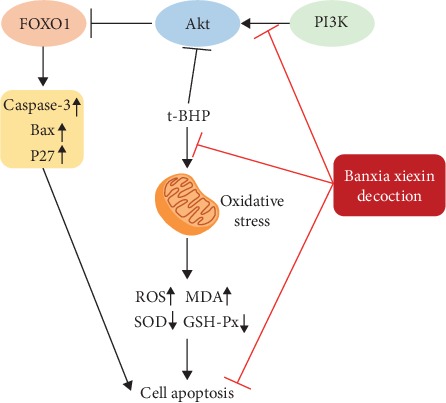
Schematic illustration of possible mechanisms of BXXD inhibited t-BHP-induced apoptosis. Protective function of BXXD for MIN6 cells against t-BHP-induced oxidative damage and apoptosis and improved insulin secretory was activation of the PI3K/AKT/FOXO1 pathway.

BXXD protected MIN6 cells against t-BHP-induced oxidative damage and apoptosis and improved insulin secretory function through modulation of the PI3K/AKT/FOXO1 pathway (Figure 8). Thus, BXXD might be developed as a therapeutic agent for T2DM.

## Data Availability

The data sets used or analyzed during the study are available from the corresponding author on reasonable request.

## Abbreviations

DM: Diabetes mellitus
T2DM: Type 2 diabetes mellitus
t-BHP: *tert*-Butyl hydroperoxide
Li: Liraglutide
LY: PI3K inhibitor LY294002
BXXD: Banxia Xiexin Decoction
ATCC: American Type Culture Collection
DMSO: Dimethylsulfoxide
DMEM: Dulbecco's modified Eagle's medium
FBS: Fetal bovine serum
PBS: Phosphate-buffered saline
MTT: 3-(4,5-Dimethylthiazol-2-yl)-2,5-diphenyltetrazolium bromide
TUNEL: Terminal deoxynucleotidyl transferase dUTP nick end labeling assay
DAPI: 4′,6-Diamidino-2-phenylindole
KRB: Krebs-Ringer bicarbonate
MDA: Malondialdehyde
SOD: Superoxide dismutase
GSH-Px: Glutathione peroxidase
ROS: Reactive oxygen species
DCFH-DA: Dichloro-dihydro-fluorescein diacetate
PBS: Phosphate-buffered saline
RIPA: Radioimmunoprecipitation assay
SDS-PAGE: Sodium dodecyl sulfate-polyacrylamide gel electrophoresis
PVDF: Polyvinylidene fluoride
TBS: Tris-buffered saline
PI3K: Phosphatidylinositol 3-kinase
AKT: Protein Kinase B
FOXO1: Forkhead box O1
Caspase-3: Cysteine aspartic acid-specific protease 3
Bcl-2: B cell lymphoma/leukemia-2
Bax: Bcl-2-associated X protein
p27: Protein 27
BCA: Bicinchoninic acid
ANOVA: One-way analysis of variance.
